# Adventitious viral genomes in vaccines but not in vaccinees.

**DOI:** 10.3201/eid0701.010124

**Published:** 2001

**Authors:** R A Weiss

**Affiliations:** University College London, London, UK.


### Adventitious Viral Genomes in Vaccines but Not in Vaccinees

It is a pleasant change to write about viruses that might have emerged but haven't. In this issue, Hussain and colleagues at the Centers for Disease Control and Prevention, the U.S. Department of Agriculture, and Harvard University report that recipients of measles, mumps, and rubella (MMR) vaccine show no evidence of infection by endogenous avian retroviruses, even though viral genomes and reverse transcriptase activity have been detected in vaccine preparations. Influenza, yellow fever, and MMR vaccines are usually prepared in embryonated eggs or in cultures of chick embryo fibroblasts (CEF). These fibroblasts contain and express endogenous retroviral genomes (1). In any vaccine, adventitious agents in the cellular substrate may contaminate the biological product. In live, attenuated vaccines, such contaminants are not inactivated, and endogenous retroviruses by their very nature as Mendelian transmitted genomes are particularly difficult to eliminate. Endogenous retrovirus release also has ramifications for pharmaceutical proteins made in cell substrates (e.g., monoclonal antibodies) and for xenotransplantation (2,3).

Some 45 years ago, it was found that apparently healthy hens could transmit avian leukosis virus (ALV) vertically in eggs (4); later it was demonstrated that live virus vaccines made in CEF were contaminated with infectious ALV (5). However, no increased risk for cancer was found in yellow fever vaccinees with the longest presumed exposure to ALV (6). Nevertheless, vaccine manufacturers were soon required to use eggs or CEF from leukosis-free flocks. To screen for ALV infection, a complement fixation for ALV (COFAL) antigen test was devised, and through pioneering work in the 1960s, the existence of endogenous retroviruses came to light because many ALV-free birds were COFAL positive (7-9).

As a graduate student at the time, I observed that CEF of COFAL-positive embryos complemented envelope-defective Rous sarcoma virus, yielding pseudotype viruses with xenotropic properties. The endogenous virus was genetically transmitted in chickens but was infectious for other hosts such as quail and pheasant. Many copies of partial or complete ALV genomes were located in chicken DNA (1). We showed that ALV had colonized the host germ line of red jungle fowl before domestication to become chickens but after divergence of the genus *Gallus* into distinct species. Even so, it proved possible in the 1970s to breed white leghorns free of endogenous ALV genomes; such chickens are now being introduced by Merck as preferred substrates for vaccine production.

A second class of endogenous avian retroviral genome (EAV), discovered in 1985 (10), is present in all breeds of chicken and cannot be eliminated. EAV can release noninfectious virus particles containing active reverse transcriptase; and this is the genome most commonly found in MMR and other vaccines (Hussain et al., this issue; 11). The major retroviral pathogen of meat-strain chickens is an infectious recombinant between ALV *gag* and *pol* genes and an *env* gene related to EAV (12). This virus has not been observed to infect human cells.

May we assume, therefore, that chicken cell substrate vaccines are safe? With biological products, as with crossing the street, there is no such thing as absolute safety. The paper by Hussain et al. is reassuring, and I agree with the authors that no change in current U.S. policies (or WHO policies, for that matter) is warranted, and the public should continue to enjoy the benefit of the vaccine. However, it may be useful to probe the possibility of interaction between endogenous avian viruses and the infectious components of MMR. We showed that vesicular stomatitis virus (VSV) could assemble its glycoprotein G on avian retrovirus virions and vice versa (13). Indeed, VSV G protein has become an envelope of choice for retroviral vectors developed for gene therapy. By analogy, the assembly of the hemagglutinin and fusion glycoproteins of measles or mumps viruses might confer a human host range on endogenous ALV or EAV particles. The possible generation of such pseudotypes or phenotypically mixed virions in vaccines may be worthy of investigation.

In addition, with ultrasensitive techniques, such as polymerase chain reaction (PCR) gene amplification, we can detect viral genomes and reverse transcriptase activity more readily in vaccine preparations. Virtually all vertebrates studied, including humans, carry endogenous retroviral genomes as part of their natural genetic constitution (1,14). Therefore, almost any cell substrate for vaccine production (avian, rodent, or primate) is likely to contain and express (at low level) endogenous retroviral genomes.

Vaccine contamination by adventitious viruses in the cellular substrate has, of course, occurred before. In one instance, the discovery of SV40 in rhesus macaque kidney cultures (15) soon led to the adoption of cynomolgus macaque and later African green monkey (AGM) kidneys as the preferred substrate for polio vaccines. That was, perhaps, a near escape as AGMs are now known to frequently harbor a strain of simian immunodeficiency virus (SIV) that luckily does not appear to infect humans. Following the potential exposure of millions of polio vaccinees to SV40, no evidence was found of increased cancer incidence (16). More recently, it has been reported that SV40 is present in some human cancers (17). Cases include pediatric tumors in patients born long after SV40 was eliminated from polio vaccines.

Ironically, it was the misguided attention of regulatory groups on hypothetical oncogenic DNA that led to vaccine contamination by adventitious oncogenic viruses in the first place. Fear of oncogenic DNA made tumor cell lines taboo as cellular substrates for vaccine production. Despite all we have learned about oncogenes and tumor suppressor genes in multistep progression to cancer, the possible trace of "oncogenic" DNA in vaccines prepared in established cell lines remained of greater concern to regulators than adventitious infections in primary cells. It is high time to reevaluate the relative risks, so it is heartening that the Food and Drug Administration held a workshop last year to begin that process.

**Robin A. Weiss**
University College London, London, UK

### References

1. Coffin J. Endogenous viruses. In: Weiss RA, Teich NM, Varmus HE, Coffin J, editors. RNA tumor viruses. New York: Cold Spring Harbor Laboratory Press;1982.p. 1109-203.
2. Weiss RA. Retroviruses produced by hybridomas. N Engl J Med 1982;307:1587.
3. Patience C, Takeuchi Y, Weiss RA. Infection of human cells by an endogenous retrovirus of pigs. Nat Med 1997;3:282-6.
4. Burmester BR, Gentry RF, Waters NF. The presence of the virus of visceral lymphomatosis in embryonated eggs of normal appearing hens. Poultry Sci 1955;34:609-17.
5. Dougherty RM, Harris RJ, Biggs PM, Payne LN, Goffe AP, Churchill AE, et al. Contaminant viruses in two live virus vaccines produced in chick cells. J Hyg 1966;64:1-7.
6. Waters TD, Anderson PS, Beebe GW, Miller RW. Yellow fever vaccination, avian leukosis virus, and cancer risk in man. Science 1972;177:76-7.
7. Dougherty RM, DiStefano HS. Lack of relationship between infection with avian leukosis virus and the presence of COFAL antigen in chick embryos. Virology 1966;29:586-95.
8. Dougherty RM, DiStefano HS, Roth FK. Virus particles and viral antigens in chicken tissues free of infectious avian leukosis virus. Proc Natl Acad Sci U S A 1967;58:808-17.
9. Payne LN, Chubb RC. Studies on the nature and genetic control of an antigen in normal chick embryos which reacts in the COFAL test. J Gen Virol 1968;3:379-91.
10. Dunwiddie C, Faras AJ. Presence of retrovirus reverse transcriptase-related gene sequences in avian cells lacking endogenous avian leukosis viruses. Proc Natl Acad Sci U S A 1985;82:5097-101.
11. World Health Organization. Reverse transcriptase activity in chicken-cell derived vaccine. Wkly Epidemiol Rec 1998;73:209-12.
12. Bai J, Payne LN, Skinner MA. HPRS-103 (exogenous avian leukosis virus, subgroup J) has an env gene related to those of endogenous elements EAV-0 and E51 and an E element found previously only in sarcoma viruses. J Virol 1995;69:779-84.
13. Weiss RA, Boettinger DE, Love D. Phenotypic mixing between vesicular stomatitis virus and avian RNA tumor viruses. Cold Spring Harbor Symp Quant Biol 1975;39:913-8.
14. Patience C, Wilkinson DA, Weiss RA. Our retroviral heritage. Trends Genet 1997;13:116-20.
15. Sweet BH, Hilleman MR. The vacuolating virus, SV40. Proc Soc Exp Biol Med 1960;105:420-7.
16. Nathanson N, Shah K. Human exposure to SV40: review and comment. Am J Epidemiol 1976;103:1-12.
17. Butel JS, Lednicky JA. Cell and molecular biology of simian virus 40: implications for human infections and disease. J Natl Cancer Inst 1999;91:119-34.
